# Individual Differences in the Perception of Color Solutions

**DOI:** 10.3390/foods7090154

**Published:** 2018-09-18

**Authors:** Ulla Hoppu, Sari Puputti, Heikki Aisala, Oskar Laaksonen, Mari Sandell

**Affiliations:** 1 Functional Foods Forum, University of Turku, 20014 Turku, Finland; ulla.hoppu@utu.fi (U.H.); sari.puputti@utu.fi (S.P.); heikki.aisala@utu.fi (H.A.); 2 Food Chemistry and Food Development, Department of Biochemistry, University of Turku, 20014 Turku, Finland; oskar.laaksonen@utu.fi; 3 Monell Chemical Senses Center, Philadelphia, PA 19104, USA

**Keywords:** color, visual, taste, perception, gender

## Abstract

The color of food is important for flavor perception and food selection. The aim of the present study was to evaluate the visual color perception of liquid samples among Finnish adult consumers by their background variables. Participants (*n* = 205) ranked six different colored solutions just by looking according to four attributes: from most to least pleasant, healthy, sweet and sour. The color sample rated most frequently as the most pleasant was red (37%), the most healthy white (57%), the most sweet red and orange (34% both) and the most sour yellow (54%). Ratings of certain colors differed between gender, age, body mass index (BMI) and education groups. Females regarded the red color as the sweetest more often than males (*p* = 0.013) while overweight subjects rated the orange as the sweetest more often than normal weight subjects (*p* = 0.029). Personal characteristics may be associated with some differences in color associations.

## 1. Introduction

Perception of food is always a multisensory experience, and the sense of sight is important for the first impression of food before tasting. Visual cues in general, such as color, shape, variety and portion size, are important determinants of food selection and acceptance [1]. For example, the color of a fruit may be an indicator of ripeness or spoilage [2]. Color may also be associated with the perceived energy content of food [3]. The use of food colorings, both synthetic and natural, is common in the food industry [4]. However, the perception of color is often neglected in the sensory evaluation of food products.

Food color may influence flavor perception, especially flavor recognition or flavor intensity [5]. Colors may be associated with taste modalities [6] and have been shown to modulate the flavor responses in different types of products, e.g., in fruit drinks [7], in fruit-flavored yoghurts [8], in sugar-coated chocolates [9] and noodles [10]. An atypical color of a food product (blue potato) has been shown to affect consumer’s perception and choice, but consumer groups may differ, for example depending on food neophobia and age [11]. Consumers may have conflicting views on the acceptance of blue food and drink [12].

In addition to the color of the product itself, the package color may also elicit associations for product flavor [13] and attractiveness [14]. Even the color of food labels may relate to consumers’ attention and product perception [15]. When serving the food, the color of cups [16] or plates [17] may influence the perception of food flavor. Even ambient light color may affect consumers’ acceptance and willingness to eat. For example, Yang et al. [18] reported that yellow light enhanced the consumption of apples. Furthermore, a colored background has been found to affect the attractiveness of fresh vegetables [19]. Spence [20] has recently reviewed the impact of background color on food perception. These examples highlight the crucial effect of color on food perception, choice and consumption.

Individual differences in the perception of chemosensory stimuli have been observed, e.g., odor perception [21]. Taste perception may be related to gene polymorphisms, gender, age and body weight [22,23]. Little is known about the background characteristics in determining the individual color associations. Interestingly, cross-cultural differences were observed in perceived connections between taste modalities and visual features [24], and therefore it is important to study colors in different countries and consumer groups. The present study evaluated Finnish adult consumers’ color perception of liquid samples by the sense of sight only. The aim was to investigate the connection of their personal background characteristics including gender, age, BMI and education on the perception of color association variables (pleasantness, healthiness, sweetness and sourness).

## 2. Methods

### 2.1. Participants

Subjects were Finnish adults (*n* = 205) who participated in a larger study with taste sensitivity tests, described in detail previously [25]. The study protocol was approved by the Ethical committee of the Hospital District of Southwest Finland and subjects gave written informed consent. Their background characteristics, including gender, age and education, were collected via electronic questionnaire before the study visit. Self-reported weight and height were used to calculate body mass index (BMI).

### 2.2. Color Solutions

Evaluation of the characteristics of the colored samples was performed in the sensory laboratory (ISO 8589) of Functional Foods Forum, University of Turku. Six different-color samples (100 mL) were presented in transparent glass bottles (V = 100 mL) with white caps and evaluated in daylight lighting. The contents of the color samples are presented in Table 1. The samples, except for the white sample, were prepared and stored for a maximum of four weeks, protected from light and at room temperature. The white sample (milk) was stored under refrigeration maximum of two days and let to settle to room temperature before evaluation. The presentation order of the samples was kept constant, as indicated in Table 1. The subjects were allowed to touch and move the bottles, but opening the caps was forbidden. The subjects were asked to rank the samples from the most to the least (ordered from 1 to 6) according to four different attributes: pleasantness, healthiness, sweetness and sourness. Additionally, the attribute presentation order was kept constant. The data was collected with Compusense five Plus software (Compusense Inc., Guelph, ON, Canada). The respondents were asked for verbal descriptions to explain their decisions for the samples in the first and last position. The descriptors were semi-quantified using NVivo 11 (QSR International Pty Ltd., Cambridge, MA, USA) software.

### 2.3. Statistics

The background characteristics of subjects are presented as proportion (%) or mean (SD). For the analysis subjects were divided into two groups based on the median age of 39 years; younger ≤ 39 years and older ≥ 40 years. The education level was grouped to lower (lower than polytechnic degree) and higher (at least polytechnic or university degree) education. BMI values classified subjects to normal weight (BMI < 25 kg/m^2^) and overweight (BMI ≥ 25 kg/m^2^).

The proportions of subjects who ranked the color as first in each attribute (the most pleasant, healthy, sweet, sour) were compared with chi-square (χ^2^) test (two groups by background variable as follows: gender: male/female, age: younger/older, BMI: normal weight/overweight, education: lower/higher education). The whole distribution of the color (from the most to the least, i.e., 1–6) in the attribute was compared by Mann–Whitney *U* test between the two groups by each background variable (gender: male/female, age: younger/older, BMI: normal weight/overweight, education: lower/higher education). *p*-value < 0.05 was considered significant and IBM SPSS 23.0 (IBM Corporation, Armonk, NY, USA) was used for statistics.

## 3. Results

Of the 205 participants, 80% were females. The age range was 19–79 years and the mean age of the subjects was 42 years (SD 15). The mean BMI was 25.6 kg/m^2^ (SD 5.6) and 44% were overweight (BMI ≥ 25). Sixty-four percent of the subjects had a high education level (polytechnic or university degree).

The frequencies of the color samples selected as the most or the least pleasant, healthy, sweet and sour are shown in Table 2 (three most common for each attribute). More than half of the subjects rated the white sample as the healthiest and the yellow as the sourest. Red and orange were rated equally often (34% both) as the sweetest. Nearly two thirds of the participants rated the blue sample as the least healthy while the white was most often considered as the least pleasant, least sweet as well as least sour (Table 2).

For some colors, it can be noticed from Table 2 that the subjects’ perceptions were at both extremes of the scale. Nearly as many subjects considered green either as the most healthy or the least healthy (14% and 16%, respectively). The yellow sample was considered the most sweet by 13% and the least sweet by 20% of the subjects. Red and orange were also regarded as the most or the least sour.

In Table 3, the significant differences in the color associations by background characteristics are presented. Females regarded the red as the sweetest more often than males (χ^2^, *p* = 0.013), while males perceived the red more often as the most healthy and the most sour. The whole distribution of the red color in those attributes also differed between genders (all Mann–Whitney *p* < 0.05). Overweight subjects regarded orange as the sweetest more often than the normal weight subjects (χ^2^, *p* = 0.029). BMI and age were related to the perception of white color, as normal weight and younger subjects regarded it as the healthiest more frequently than overweight and older subjects (Table 3).

In addition, the older subjects regarded the green colored sample as the healthiest more often than the younger subjects (19% vs. 9%; χ^2^ *p* = 0.042), but the entire green–healthy distribution did not differ. Considering educational level, the less-educated participants selected the green as the most healthy more frequently than the higher-educated participants (24% vs. 9%, *p* = 0.004) as well as the orange as the most sour less frequently (5% vs. 15%, *p* = 0.029). Regarding pleasantness, no significant differences in color pleasantness were observed between background characteristic groups.

The discrepancy between the evaluations of red, orange and yellow colors as the most sweet or sour was focused in more detail based on the qualitative descriptions by the subjects. The descriptive terms most often used as the basis of selecting the color sample as the sweetest were as follows: in the case of red—juice, candy, berries, strawberry; for orange—juice, berries, candy; and for yellow—honey and candy. On the other hand, the descriptive terms for these colors when chosen as the most sour were as follows: red—berry juice/juice, lingonberry, cranberry; orange—berry juice/juice, berried (sour berries); yellow—lemon/citrus. These descriptions highlight the individual differences in color perception; e.g., red color can be regarded as either sweet or sour and is associated with different food items.

## 4. Discussion

In the present study, the color associations varied considerably and no single color could be regarded as explicitly related to only one attribute. There are few previous findings on the background characteristics of subjects in relation to color associations. In this study, gender, weight status, age and education level associated with differences in the perception of specific colors. For professionals working with product development, consumer behavior and sensory evaluation of food, it is important to understand how various demographic and other individual characteristics of consumers may affect their responses.

Spence et al. [26] have reviewed previous studies on the association of certain colors to basic tastes, and they concluded that sweet taste is most often associated with red or pink and sour to green or yellow. Recently in Australia, Saluja and Stevenson [27] reported very similar associations and they also found that the saturation of color choices was related to tastant concentration. In the present study, red and orange were chosen as the most sweet equally often (34% both) and yellow as the most sour by more than half of the subjects. However, many subjects also regarded red or orange as the sourest, indicating a large variation in color perception. Only color perception related to sweetness and sourness was studied, and thus in future it would be interesting to determine Finnish consumers’ perception of colors in relation to salty and bitter tastes. Color pairs have also been compared with single colors in color taste associations, but the color pairs took more time to match to tastes [28].

Zampini et al. [29] have investigated different colors related to flavor discrimination and flavor intensity ratings in fruit-flavored solutions. They demonstrated that solutions of certain colors are more often associated with particular flavors, e.g., yellow-colored solutions with lemon flavor and green with lime flavor. Furthermore, they found that flavor discrimination was impaired by coloring solutions inappropriately [29]. Wan et al. [30] reported that the type of receptacle (water glass, wine glass, cocktail glass or plastic cup) modulated the expected flavor of a colored beverage. Color–flavor associations may be product-specific and have often been studied in beverages. In orange juice samples, color changes did not affect flavor intensity or sweetness, but a greenish hue significantly increased perceived sourness [31]. The context of the color samples and the color hue may affect the perceptions, and thus the associations found in the present study may be affected by the hue of the liquid samples. Also, the liquid form of the samples may contribute to different associations compared to solid samples.

Qualitative answers gave new insight into the individuality of color associations. Because Finnish consumers’ color perceptions have not been studied before, the open-answer option for color explanations was used instead of ready lists to select color–flavor associations. Interestingly, the answers also highlight the role of berries in Finnish food culture. Sweetened and unsweetened berry juices, both home-made and industrial, are frequently consumed in Finland, and thus it seems that consumers have different associations of red and orange color to berry or berry juice sweetness or sourness. Color–flavor associations may be culture-specific, as observed in previous studies [24]. Different expectations may explain cultural differences in color–flavor associations [32].

Regarding gender differences in color perception in the present study, interestingly, the perception of red color differed. Females perceived red as the sweetest more often than males while the opposite was observed for sourness. Gender differences in color–flavor associations have seldom been studied previously. However, it has been observed that at breakfast under different lighting, blue lighting decreased the amount of food consumed in men but not in women [33]. Generally, it has been concluded that women may be more reactive to visual food stimuli [34]. A novel finding was also that the perception of orange color in relation to sweetness and sourness differed between the normal weight and overweight people. Considering the high prevalence of overweight in Finland nowadays, it is important to take into consideration that weight status may affect color and food perception.

The white sample was regarded as the healthiest most often, but there were differences between BMI and age groups. Many subjects associated white-colored liquid to milk, which is commonly recommended and consumed in Finnish food culture. Perceptions of color healthiness may also be product or situation-specific. In an approach–avoidance task with healthy and unhealthy food items, it was found that red color supported avoidance reactions to unhealthy foods [35]. The color red in a plate or cup has been shown to reduce snack food and soft drink intake [36]. Colorfulness in general may be associated with healthy food choices, and König and Renner [37] reported that the increased color variety of a meal was related to the increased intake of vegetables. More research is needed to understand the potential of colors to promote healthy food choices.

This study gave important new information regarding the color perception of Finnish consumers, but further studies are needed to understand the complex interactions between senses. The tested color solutions were evaluated by the sense of sight only, but regarding the multisensory experience of food, odor–color interactions should also be evaluated [38]. The consumers’ expectations and perception of food color may be mediated by emotion, as emotion terms have been shown to be associated with certain colors [39]. Considering study scheduling, it should be noted that there may be temporal differences in color evaluations; for example, Schloss and Heck [40] reported seasonal changes in color preferences.

As a whole, both intrinsic and extrinsic product characteristics affect preferences [41]. In conclusion, individual color perceptions and preferences may vary significantly, emphasizing the need for further studies to understand color perception and its connection to choices in different consumer segments.

## Figures and Tables

**Table 1:** Color solutions.

Presentation Order	Color	Preparation
1	Orange	2.5 mg Chromotrope FB + 45 mg Quinoline Yellow/100 mL water
2	White	Milk, fat content 1.5% (UHT, Valio, Helsinki, Finland)
3	Blue	2.5 mg Patent Blue V, sodium salt/100 mL water
4	Yellow	50 mg Quinoline Yellow/100 mL water
5	Green	25 mg Quinoline Yellow + 1.25 mg Patent Blue V, sodium salt/100 mL water
6	Red	25 mg Chromotrope FB/100 mL water

Chromotrope FB, CAS 3567-69-9, Alfa Aesar GmbH & Co KG (Karlsruhe, Germany); Quinoline Yellow, CAS 8004-92-0, Acros Organics (New Jersey, NJ, USA); Patent Blue V, sodium salt, CAS 20262-76-4, Acros Organics.

**Table 2:** Three most frequently (columns 1., 2., 3.) selected color samples as the most or the least pleasant, healthy, sweet and sour (% of subjects).

	1.	2.	3.
Most pleasant	Red 37%	Orange 22%	White 14%
Least pleasant	White 29%	Yellow 22% Green 22%	Blue 20%
Most healthy	White 53%	Red 22%	Green 14%
Least healthy	Blue 65%	Green 16%	Yellow 13%
Most sweet	Red 34% Orange 34%	Yellow 13%	Green 9%
Least sweet	White 49%	Yellow 20%	Blue 19%
Most sour	Yellow 54%	Red 24%	Orange 11%
Least sour	White 67%	Red 8% Orange 8%	Blue 7%

**Table 3:** Significant differences in color associations by background characteristics.

	% Subjects Selecting as 1st in the Attribute	*p* Chi-Square *	*p* Mann–Whitney **
**Gender**			
Red–healthiness	Females 17% Males 40%	0.001	0.008
Red–sweetness	Females 39% Males 18%	0.013	0.020
Red–sourness	Females 19% Males 40%	0.006	0.007
**BMI**			
White–healthiness	Normal weight 61%, overweight 45%	0.029	0.06
Orange–sweetness	Normal weight 28% Overweight 43%	0.029	0.024
Orange–sourness	Normal weight 15% Overweight 5%	0.022	0.035
**Age**			
White–healthiness	Younger 61% Older 45%	0.024	0.028

*, Chi-square comparing the proportions of subjects selecting the color as the first in the attribute (as the most healthy/sweet/sour respectively) between the two background variable groups (gender: male/female, BMI: normal weight/overweight, age: younger/older); **, Mann–Whitney comparing the whole distribution of the color in the attribute (from most to least healthy/sweet/sour respectively) between the two background variable groups.
